# Effects of Modifiable Activity-Related Health Behaviors on the Sleep-Pain Relationship in Adolescents

**DOI:** 10.1007/s10880-024-10017-5

**Published:** 2024-05-09

**Authors:** Nuria Morales, Tori R. Van Dyk

**Affiliations:** Department of Psychology, Loma Linda University, 11130 Anderson Street, Suite 106, Loma Linda, CA 92354 USA

**Keywords:** Pain, Sleep, Physical activity, Screen time, Adolescents

## Abstract

Poor sleep and chronic pain are commonly related in adolescents. Only 5% of adolescents meet recommendations for physical activity and screen time, both of which impact the experience of sleep and pain disturbances. Research is needed to better understand the sleep-pain relationship in adolescents and to identify potential protective factors, such as activity-related health behaviors. This study examined sleep, behaviors that influence activity (i.e., physical activity, screen time), and their interaction as predictors of pain in a sleep-disordered sample of 105 adolescents aged 12–18 presenting for polysomnography. A hierarchical multiple linear regression was conducted to examine these relationships. Consistent with hypotheses, worse insomnia predicted worse pain. However, other activity-related health behaviors did not influence this relationship, *p*s > .05. Findings suggest that sleep should be the focus of treatment for adolescents with primary sleep disorders to prevent the onset or exacerbation of pain.

## Introduction

It is estimated that the prevalence of chronic pain for youth under the age of 18 is as high as 88% (King et al., 2011), with 5–8% experiencing severe and disabling chronic pain (Palermo et al., 2019). Youth who experience persistent pain are at risk for developing internalizing and externalizing symptoms, functional declines, decreased quality of life, and a poor trajectory of worsening pain symptoms during adolescence and into adulthood (Noel et al., 2016; Perquin et al., 2000). Considering the negative consequences associated with pain, it is important to identify factors that may contribute to the development or exacerbation of the pain experience. Poor sleep has been identified as one such factor (Lewandowski et al., 2010). However, it is possible that additional modifiable health behaviors, such as physical activity and sedentary behavior, may influence pain, sleep, and/or the sleep-pain relationship.

The relationship between sleep and pain has been well-established (Finan et al., 2013; Krietsch et al., 2021; Lewandowski et al., 2010). More than 50% of adolescents with chronic pain have sleep concerns (Palermo et al., 2011), including difficulty falling asleep, recurrent night and early morning awakenings, short sleep duration, and excessive daytime sleepiness (Badawy et al., 2019; Palermo & Kiska, 2005). These high rates are problematic as adolescents with comorbid disturbed sleep and chronic pain appear to have worse physical, social, and emotional well-being relative to adolescents with chronic pain but fewer sleep disturbances (Palermo & Kiska, 2005).

While sleep has been more extensively studied in chronic pain samples, less is known about the pain experience of youth presenting with a primary sleep disturbance. However, in the research that does exist, somatic and pain complaints appear to be common. In one study, parents reported that 61.1% of adolescents with insomnia had at least one somatic complaint with variable rates of specific pain complaints such as headaches (66.6%), stomachaches, (48.5%), and aches/pains (45.1%). In addition, greater parent-reported somatic and pain complaints were related to worse insomnia severity at intake (Van Dyk et al., 2022). While the overlap between populations is likely large, these findings suggest slightly higher rates of comorbid pain symptoms reported among adolescents with a primary sleep concern relative to the proportion of comorbid sleep disturbances reported among adolescents presenting with chronic pain.

The sleep-pain relationship is understood as bidirectional and reciprocal, in that pain can interfere with sleep by delaying sleep onset and interfering with the stability and quality of sleep (Lewin & Dahl, 1999). Likewise, the psychological and physiological effects of insufficient sleep (including worry and a decreased sense of control) can have detrimental effects on pain management (Lewin & Dahl, 1999). While sleep and pain impact one another, research has found that sleep impairments may be a stronger, more dependable predictor of pain than vice versa (Finan et al., 2013). For example, Lewandowski et al. (2010) found significant associations between total sleep time and wake after sleep onset with next-day pain reports among adolescents with chronic pain, but pain did not prospectively predict any measure of sleep. However, more research is needed to better understand the occurrence of pain in those presenting with sleep problems.

While evidence-based behavioral strategies to improve sleep exist (de Bruin et al., 2015), it is possible that targeting numerous health behaviors simultaneously may confer the greatest benefit with regard to preventing or reducing the experience of pain in youth with disordered sleep. One promising component of interventions for adults with chronic pain appears to be targeting health behaviors common among these individuals, including sedentary behavior, stress, poor sleep, and unhealthy diet (Anderson et al., 2016). All of these factors are associated with chronic pain severity and maintenance (Nijs et al., 2020), which suggests that promoting healthy behaviors may play an important role in preventing and managing chronic pain. However, most of the research investigating these health behaviors focuses on adults, resulting in little knowledge as to the role these behaviors play in the pain experience of youth. It is possible that health behaviors could impact adolescents’ pain differently due to factors such as increased autonomy and fewer structured opportunities to engage in physical activity in adulthood than in adolescence. Further, these health behaviors appear to be related to sleep in that less physical activity and high amounts of sedentary behavior (i.e., screen time) have been found to exacerbate the severity of sleep disturbances in adolescents (Foerster et al., 2019; Kline, 2014).

While there may be many health behaviors that can influence the sleep-pain relationship, the present study focuses on physical activity and screen time. The American Academy of Pediatrics recommends that adolescents sleep eight to ten hours a night, engage in 60 min of moderate or vigorous-intensity physical activity per day; and limit screen time to less than two hours within a 24-h period (Hirshkowtiz et al., 2015; Munth, 2020; Reid Chassiakos et al., 2016). However, only 5% of adolescents meet recommendations for sleep, physical activity, and screen time concurrently (Knell et al., 2019). Given that adolescents typically have inadequate levels of physical activity and high levels of screen time, which are related to both sleep and pain disturbances (Foerster et al., 2019; Hakala et al., 2012; Kline, 2014; Nijs et al., 2020), these behaviors are particularly important to consider within the context of the sleep-pain relationship.

Youth with a sleep disturbance are at heightened risk of having low levels of physical activity in that a bidirectional relationship between exercise and sleep is well-established (Kline, 2014). In experimental studies, adolescents demonstrate significantly more sedentary behavior when experiencing short versus extended sleep (Van Dyk et al., 2018). Lack of physical activity may also contribute to a greater pain experience in youth (Long et al., 2008), while increased physical activity is associated with significantly lower levels of self-reported pain intensity (Kashikar-Zuck et al., 2010). Aerobic exercise may be related to pain intensity through its influence on physiological pathways that produce nociception. For example, regular aerobic exercise increases serotonin and endogenous opioid levels, which are biological buffers of chronic pain (Lima et al., 2017). Adolescents with chronic pain have been found to engage in lower levels of physical activity compared to healthy adolescents (Long et al., 2008). Regarding the temporal relationship between the two, a bidirectional relationship likely exists, in that higher pain intensity is associated with lower next-day physical activity levels while higher physical activity levels are predictive of lower pain intensity ratings the next day (Rabbitts et al., 2014). Thus, there is evidence for a bidirectional relationship between physical activity and sleep disturbances as well as between physical activity and pain. However, more research is needed to understand how physical activity influences the sleep-pain relationship.

Another activity-related health behavior that is potentially relevant to the sleep-pain relationship is screen time. Screen time is considered a sedentary behavior that is associated with various adverse health outcomes in adolescents, including increased sleep problems (Baiden et al., 2019; Foerster et al., 2019; Odgers et al., 2020). In addition to being related to and exacerbating sleep disturbances in youth, screen time also appears to be related to pain, although this relationship is more inconsistent. For example, high amounts of computer usage have been shown to increase musculoskeletal pain among adolescents (Hakala et al., 2012) but other research has found screen time to be significantly associated with pain among adolescents only when examined in isolation, with the association becoming insignificant when taking other factors like short sleep and moderate physical activity into account (Silva et al., 2017). The latter finding suggests that it is important to consider health behaviors in conjunction as opposed to in isolation. Given that the evidence for and against a relationship between screen time and pain is scant, its inclusion in the present study may help to illuminate whether screen time is a significant predictor of pain in youth.

Sleep, physical activity, and screen time all appear to impact the development and experience of pain, although little is known about the concurrent relationships between these variables, particularly among youth. Unlike most existing literature that draws from youth with a primary pain concern, the present study uses a sample of adolescents with a primary sleep disturbance who may or may not have comorbid pain. Prior research suggests that youth who present with sleep disturbances are significantly more likely to develop pain (Simpson et al., 2018) or, if already present, have worse pain (Lewandowski et al., 2010). While it is possible to address sleep and pain concerns separately, it is also possible that by optimizing various health behaviors, the severity of sleep disturbance may decrease while simultaneously decreasing the experience of pain or the likelihood of developing pain. However, the influence of activity-related behaviors on the sleep-pain relationship has not yet been examined.

The present study will fill this gap in the literature by examining physical activity and screen time as potential moderators of the sleep-pain relationship in a sample of adolescents presenting to an overnight polysomnography with pre-existing sleep concerns. It was hypothesized that there would be a significant positive relationship between insomnia severity and pain, such that adolescents with worse insomnia would report experiencing higher levels of pain. In addition, it was hypothesized there would be a significant relationship between physical activity and screen time and an adolescent’s insomnia and pain severity such that lower physical activity and greater screen time would be associated with worse insomnia and pain. Lastly, it was hypothesized that the strength of the relationship between insomnia and pain would be significantly influenced by adolescents’ physical activity and screen time, such that the association of worse insomnia and increased pain would be strongest for adolescents with a low level of physical activity and increased screen time.

## Method

### Participants

Participants included 105 adolescents ages 12–18 and their accompanying parent/legal guardian who presented to a sleep disorders center in the United States for an overnight sleep study (polysomnography; PSG). Eligible participants included those who were referred for PSG to assess a potential or established sleep disturbance. Both the parent and child were required to speak and understand English in order to complete the study protocol.

### Procedure

Data collection at the sleep disorders center occurred between August 2019 and March 2020, and again from January 2022 to June 2023, with a pause in data collection due to the COVID-19 pandemic. The sleep disorders center is an outpatient clinic where sleep medicine physicians diagnose and provide treatment recommendations via the use of PSG to patients with sleep difficulties. At the beginning of a PSG appointment, patients and families were invited to participate in the study and parental and adolescent consent was obtained for those interested. The parent and child proceeded to independently complete tablet-based surveys asking about demographics, mental health, physical health, and sleep behavior. Thus, results from the present study are from a larger study that screened a convenience sample of adolescents presenting with sleep difficulties. Research assistants were available to ask questions and support questionnaire completion. Families were invited to enter a raffle to win an Amazon gift card ranging from $25–100. All procedures were approved by the university’s Institutional Review Boards.

### Measures

#### Pediatric Insomnia Severity Index (PISI)

Adolescents completed the PISI (Byars et al., 2017). The PISI is a six-question, self-report measure of insomnia severity. Participants were instructed to use the past week as a reference for answering questions related to how many nights per week they have difficulty falling asleep, trouble maintaining sleep, and daytime sleepiness, in addition to average sleep duration. Participants rated their behavioral frequency on a 5-point Likert scale that ranged from 0 to 5 for each statement. Option choices included: (0) 0 nights per week, (1) 1–2 nights per week, (2) 2–3 nights per week, (3) 4–5 nights per week, (4) 5–6 nights per week, and (5) 7 nights per week. Options for the question asking about hours of sleep per night were: (0) less than 5 h, (1) 5–7 h, (2) 7–8 h, (3) 8–9 h, (4) 9–11 h, and (5) 11–13 h. Total scores range from 0 to 30, with higher scores representing greater insomnia severity. The PISI has demonstrated acceptable validity through correlation with other validated sleep measures (*r* = .42) and high internal consistency (*α* = 0.80; Byars & Simon, 2014). Reliability in the present sample was good for the PISI, *α* = 0.84.

#### Pain Experience

Adolescents responded to four items concerning their pain experience via the quality of life in neurological disorders (NeuroQoL) version of the patient-reported outcomes measurement information system (PROMIS; Lai et al., 2012). Participants responded to the items using a 5-point Likert scale with responses that ranged from (0) Never to (4) Almost Always and were asked to use the past seven days as a frame of reference. Items included, “I had a lot of pain,” “I had so much pain that I had to stop what I was doing,” and “I had trouble watching TV when I had pain.” Options for the last question asking about pain duration included (0) Few seconds, (1) Few minutes, (2) Few hours, (3) Few days (less than a week), and (4) More than a week. These items were used to determine both patients’ pain severity and the impact of their pain on their functioning, which are two clinically meaningful aspects of pain. Total scores ranged from 0 to 16, with higher scores indicating a worse pain experience. Previous studies have established excellent internal reliability for the pain domain of the NeuroQoL, (*α* = 0.95; Lai et al., 2012). Reliability in our sample was good for the pain items, *α* = 0.86.

#### Physical Activity

Adolescents responded to a question that asked, “On average, how many days of the week do you participate in physical activity that makes you sweat or breathe hard for at least 30 min?” Option choices included: 1, 2, 3, 4, 5, 6, or 7 days of the week. This question has been used in prior research (Boone, 2015).

#### Screen Time

Adolescents responded to a question that asked, “In general, how many HOURS per DAY (outside of the school setting) do you usually spend watching a screen (e.g., television, tablet, computer)?” The question was designed in an open format where adolescents were able to fill in their response. This question has been used in prior research (Boone, 2015).

### Analytic Plan

First, preliminary analyses were conducted to test for the presence of outliers, missing data, and assumptions of the hierarchical multiple linear regression analyses (i.e., multicollinearity, normality). Preliminary analyses indicated missing data rates ranged from 3.8 to 20%. Considering the high rates of missing data for some variables, missing data were imputed using Bayesian imputation, which assumes the data are missing at random (Parker et al., 2022). An iterative Markov chain Monte Carlo (MCMC) algorithm was used to estimate, or impute, missing values (Metts et al., 2022). Potential scale reduction factor diagnostics and trace plots were used to determine the adequate number of burn-in cycles needed for the MCMC algorithm to converge prior to interpreting results (Gelman & Rubin, 1992). The number of effective sample sizes was assessed to determine the necessary number of post-burn-in iterations (Gelman et al., 2014). This resulted in two MCMC chains being specified with 10,000 burn-in cycles, followed by 10,000 post-burn-in iterations.

To address the primary study aims, descriptive statistics were conducted for demographics, insomnia, pain, physical activity, and screen time. Bivariate correlations were then conducted to examine relationships among insomnia severity, pain, physical activity, and screen time. Given the relatively small sample and concerns related to power, demographics were not systematically entered in the full models as covariates. Instead, correlations were examined between primary variables and demographics to identify any potential covariates; however, there were no patterns of significance, and thus no demographic covariates were included in further analyses so that power was retained. Finally, to examine physical activity and screen time as possible moderators of the sleep-pain relationship, a hierarchical multiple linear regression was used to examine insomnia severity (entered on the first step) and modifiable activity-related health behaviors including physical activity and screen time (entered on the second step) as individual predictors of pain. The physical activity x insomnia interaction and screen time x insomnia interactions were entered on the third step of the model to examine the moderating effect of each health behavior on the association between insomnia and pain. Following procedures recommended by Enders (2022), all variables used in each specific model were entered simultaneously and informed imputation.

Based on the linear multiple regression analyses, an a priori power analysis was conducted using G*Power (Faul et al., 2009). The power analysis estimated a need for 92 participants to have 80% power to detect a significant medium effect size (*f*^2^ = 0.15) at an alpha of 0.05 with five predictors (i.e., insomnia severity, physical activity, screen time, the interaction between physical activity and insomnia severity, and the interaction between screen time and insomnia severity). Existing literature examining relationships between primary variables has found effect sizes ranging from small (Van Dyk et al., 2022) to large (Van Dyk et al., 2018). Because of the variability in effect sizes, a medium effect size was selected.

RStudio Version 1.3.1093 was used to test for assumptions of the hierarchical multiple linear regression analyses (RStudio Team, 2020). SPSS Version 28 was used to conduct descriptive analyses on demographic variables of interest (IBM Corp., 2021). All descriptives for primary variables and bivariate and regression analyses were conducted using Blimp 3 (Keller & Enders, 2021). Blimp uses Bayesian estimation, which provides information on posterior median estimates of parameters and their associated 95% credible intervals (95% Crls). Credible intervals are intuitively similar to frequentist confidence intervals in that they represent the probability that the true effect lies within the specified interval (Joiner et al., 2022). Consequently, a 95% CrI that does not include zero is interpreted as representing a statistically significant effect at *p* < .05.

## Results

### Descriptive Statistics

The present sample consisted of 105 adolescents between the ages of 12 and 18 (*M* = 14.62, *SD* = 1.74), 50.5% of whom identified as male. The adolescent participants represented ethnically diverse backgrounds; per parent report, a majority (*N* = 55) identified as White and Hispanic/Latinx (62.5%). However, racial diversity in the present sample was limited, with only 15.8% of participating adolescents identifying as a race other than White. While the average annual household income for the present sample was mixed, a majority (*N* = 55) fell below $50,000 (51.4%). The median annual income for the local community is $55,372 (U.S. Census Bureau, 2017–2021). A board-certified sleep physician diagnosed organic sleep disorders based on PSG findings. Results on the evening of data collection indicate that 58% of adolescent participants had one or more diagnoses with OSA being the primary diagnosed sleep disorder. Refer to Table 1 for the full demographics of participating adolescents.

**Table 1:** Demographics of participating adolescents (*N* = 105)

Demographics	*M* (*SD*); *N* (%)
Youth’s age	14.65 (1.73)
Youth’s sex	
Male	53 (50.5%)
Female	52 (49.5%)
Ethnicity	
White, Hispanic/Latinx	55 (62.5%)
White	15 (17.0%)
Black	4 (4.5%)
Asian	3 (3.4%)
Black, Hispanic/Latinx	3 (3.4%)
Asian, Hispanic/Latinx	2 (2.3%)
White, Asian	2 (2.3%)
Middle Eastern	1 (1.1%)
White, Black	1 (1.1%)
White, Native Hawaiian	1 (1.1%)
American Indian	1 (1.1%)
Annual household income	
Under $10,000	12 (14.0%)
$10,000–19,999	10 (11.6%)
$20,000–29,999	11 (12.8%)
$30,000–39,999	11 (12.8%)
$40,000–49,999	10 (11.6%)
$50,000–74,999	14 (16.1%)
$75,000–99,999	4 (4.7%)
$100,000–150,000	9 (10.5%)
Over $150,000	5 (5.8%)
Organic sleep disorders	
Obstructive sleep apnea	63 (63.0%)
Central sleep apnea/hypopnea	9 (9.0%)
Restless leg syndrome	14 (14.0%)
Sleep related bruxism	3 (3.0%)
Sleep related hypoventilation	1 (1.0%)
Periodic limb movement disorder	1 (1.0%)
Number of organic sleep disorders	
0	25 (25.0%)
1	58 (58.0%)
2+	17 (17.0%)

Differences in sample size are due to missing data

In the present sample, 44.8% of youth indicated they experienced “a lot of pain” in the past week “sometimes” to “almost always” and reported an average total pain score of 4.42 (*SD* = 4.28) out of a possible 0–12 score range. The average level of insomnia for the present sample of adolescents was 13.79 (*SD* = 7.55) out of a possible 0–30 score range. Regarding health behaviors, adolescents reported spending an average of 4.82 h of screen time per day outside of the school setting (*SD* = 2.67) and an average of 4 days of physical activity per week (*SD* = 2.00). Refer to Table 1 for detailed information regarding adolescent demographics and Table 2 for descriptive statistics for primary variables.

**Table 2:** Descriptive statistics for primary variables

	*M*	*SD*	Min^a^	Max^a^
Pediatric insomnia severity index	13.79	7.55	0	30
Pain	4.42	4.28	0	12
Physical activity	4.17	2.00	1	7
Screen time	4.82	2.67	1	10

^a^Values represent possible minimum and maximum for each given measure, with the exception of screen time which was an open-ended question and values represent the minimum and maximum of the observed data

### Bivariate Analyses

Bivariate analyses revealed higher levels of insomnia to be correlated with greater levels of pain (*r* = .36, *p* < .05). Physical activity and screen time were not found to be significantly correlated with pain, insomnia, or with one another (*p*s > .05). Refer to Table 3 for bivariate correlations between primary variables.

**Table 3:** Bivariate correlations between primary variables

Variables	PhyAct	ScrTime	Pain
PISI	− 0.03	0.18	0.36*
PhyAct	–	− 0.12	− 0.18
ScrTime		–	0.13

*PISI* pediatric insomnia severity index, *PhyAct* physical activity, *ScrTime* screen time

*p < .05

### Regression Analyses

To evaluate the effect of activity-related modifiable health behaviors (i.e., physical activity and screen time) on the sleep-pain relationship, a stepwise hierarchical multiple linear regression was conducted. Insomnia was entered as an individual predictor of pain on step 1 of the model and significantly accounted for 12.6% of the variance in pain. As insomnia severity increased by one point, pain increased by 0.20 points, *p* < .05. Physical activity and screen time were added on the second step of the model to examine their individual effects on pain, and while this model accounted for an additional 4.4% of the variance in pain, none of the aforementioned health behaviors were significantly associated with pain above and beyond the effects of insomnia, *p*s > .05. Specifically, the addition of physical activity and screen time did not account for a significant proportion of additional variance in pain, *X*^2^ (3) = 2.61, *p* = .27. The interactions between physical activity and screen time with insomnia were entered on the final step and while this model accounted for an additional 4.3% of the variance in pain, this was not a statistically significant increase, *X*^2^ (3) = 3.10, *p* = .21 (see Table 4). However, insomnia remained a significant predictor of pain on steps two and three of the model, such that as insomnia severity increased by one point, pain increased by 0.20 points, and by 0.20 points, respectively. In summary, while insomnia severity was associated with pain in adolescents, the addition of physical activity, screen time, and their interactions with insomnia did not account for variance in pain above and beyond the effect of insomnia, *p*s > .05.

**Table 4:** Results of a hierarchical linear regression predicting pain from average insomnia, physical activity, screen time, and their interactions

Predictor	*R*^2^_adj_	Δ*R*^2^	*b*	*β*	95% CrI
Full model	0.21	0.04			
PISI			0.20	0.34	[0.08, 0.32]
PhyAct			− 0.34	− 0.15	[− 0.80, 0.14]
ScrTime			0.09	0.06	[− 0.23, 0.41]
PISI × PhyAct			− 0.06	− 0.20	[− 0.13, 0.01]
PISI × ScrTime			− 0.02	− 0.07	[− 0.27, 0.13]

*PISI* pediatric insomnia severity index, *PhyAct* physical activity, *ScrTime* screen time

## Discussion

The present study aimed to better understand how modifiable activity-related health behaviors (i.e., physical activity, screen time) impact the sleep-pain relationship among adolescents presenting with sleep difficulties. To understand the implications of the present findings, it is important to contextualize the present sample. Adolescents in our sample reported an average insomnia severity of 13.79 which is lower than other comparable samples. Specifically, the average insomnia severity score on the same scale for a separate adolescent sample presenting with a primary diagnosis of insomnia to an outpatient behavioral sleep medicine clinic was 18.22 (Van Dyk et al., 2019). The relatively less severe insomnia symptoms in the present sample may be because adolescents were presenting for diagnostic sleep testing for a variety of sleep disorders beyond just primary insomnia (e.g., obstructive sleep apnea). Specifically, 58% of participating adolescents were diagnosed with at least one organic sleep disorder. However, despite our sample drawing from a broader range of possible sleep disorders, rates of experiencing pain appear to be similar to this comparable, insomnia-specific adolescent sample. Specifically, approximately 45% of our sample of adolescents indicated they experienced pain in the past week which is a nearly identical rate to the proportion of adolescents endorsing experiencing aches/pains in the insomnia-specific sample (Van Dyk et al., 2022). This indicates that pain rates may be similar among a variety of sleep disorders, including both those with a primary diagnosis of insomnia and/or an organic sleep disorder.

Regarding health behaviors, adolescents in our sample reported an average daily screen use of approximately five hours outside of school, which exceeds the recommended two hours or less within 24 h but is in line with prior literature suggesting adolescents have high rates of screen usage (Nagata et al., 2022; Reid Chassiakos et al., 2016). Further, adolescents reported engaging in physical activity (defined as participating in physical activity that makes you sweat or breathe hard for at least 30 min) approximately four times per week on average, which corroborates prior findings that adolescents do not meet the recommended sixty minutes of daily physical activity (Munth, 2020). In summary, the health behaviors of the adolescents in the sample are by and large representative of the trend of poor engagement in activity-related health behaviors among adolescents and indicate that adolescents are regularly not meeting the American Academy of Pediatrics guidelines (Munth, 2020; Reid Chassiakos et al., 2016).

Consistent with our hypothesis, we found that insomnia and pain were correlated with one another and that worse insomnia severity was predictive of higher levels of pain. However, contrary to hypotheses, physical activity and screen time were not significantly related to insomnia or pain severity. Further, our hypothesis that the strength of the relationship between insomnia and pain would be significantly influenced by adolescents’ activity-related health behaviors was not supported.

The finding that insomnia and pain are related is consistent with prior literature (Palermo et al., 2011; Van Dyk et al., 2022) and supports the notion that sleep impairments are associated with worse pain (Lewandowski et al., 2010). While much of the existing literature examining the sleep-pain relationship in youth focuses on pain samples (e.g., Boggero et al., 2021; Logan et al., 2015; Palermo et al., 2007), the present study furthers our understanding by providing evidence for considerable rates of co-morbid pain among a sample of adolescents with a primary sleep concern. There are likely neurobehavioral, functional, and cognitive levels mechanisms maintaining the sleep-pain relationship. For example, dopaminergic pathway disturbances and opioidergic signaling have been implicated as biobehavioral mechanisms behind this relationship (Finan et al., 2013). Dopamine plays a crucial role in arousal and has been found to be altered by pain, while endogenous opioid systems become dysregulated by sleep deprivation, in turn impacting the efficacy of opioid analgesic properties (Finan et al., 2013). In addition, there are similarities in the cognitive profiles of adolescents with sleep and pain disturbances, such that catastrophizing thoughts about sleep, pain, and pain disability may underpin the observed comorbidity (Palermo et al., 2011). The neurobehavioral, functional, and cognitive similarities underlying sleep and pain may help to explain why similar rates of pain are observed among a variety of sleep disorders (e.g., behavioral insomnia, organic sleep conditions).

This study was the first of its kind to examine modifiable activity-related health behaviors (i.e., physical activity, screen time) in the context of the insomnia-pain relationship among adolescents. However, these behaviors did not seem to matter above and beyond the impact of insomnia. Mechanistically, the overlap in the underlying neurobehavioral systems for pain and sleep regulation, such as the impact of stress on both pain and sleep disturbances, as well as the deteriorative effects of insufficient sleep on pain habituation and sensitization, may help to explain why sleep plays a crucial role in the development or exacerbation of pain (Lewin & Dahl, 1999; Simpson et al., 2018). While the aforementioned health behaviors have been suggested to impact sleep disturbances and chronic pain severity and sustainment independently (Nijs et al., 2020), the mechanisms underlying the sleep-pain relationship may be more important. Additionally, poor sleep has been found to directly impact activity-related health behavior, such as sedentary behavior (Van Dyk et al., 2018). For example, while regular aerobic exercise is associated with increased serotonin and endogenous opioid levels, which are biological buffers of chronic pain (Lima et al., 2017), it is possible that poor sleep dysregulates endogenous opioid systems, which may disrupt the positive effects of physical activity on pain. However, more research is needed to examine these relationships and underlying mechanisms.

Clinically, the present findings support the notion that improving sleep may prevent the development or exacerbation of pain in adolescents presenting with sleep disturbance. The same may be true for youth with pain, as the sleep-pain relationship appears to be bidirectional, suggesting it is important to consider sleep and pain simultaneously (Kline, 2014). While addressing sleep disturbances in youth, it should be acknowledged that a large proportion of these youth experience pain regularly, and by addressing pain and sleep concerns simultaneously, further downstream negative consequences (e.g., anxiety, depression; Noel et al., 2016) may be prevented. Further, if pain symptoms go ignored, the presence of pain may exacerbate sleep disturbances, for example, through high levels of cognitive arousal at bedtime (Palermo et al., 2011), and/or may serve as a barrier to effective sleep treatment if not incorporated. For example, chronic pain is associated with increased sedentary behavior and rest during pain spikes (e.g., lying in bed during a pain episode or trying to sleep off a headache), which, from a stimulus control perspective (e.g., associating the bed with arousal as opposed to sleep), can negatively impact one’s ability to fall and stay asleep. Incorporating information about an adolescent’s comorbid pain, such as the use of naps while in pain, can be used to inform insomnia treatment to increase its efficacy.

A strength of the present study is that it draws on a unique, ethnically diverse sample of adolescents presenting with a wide range of suspected sleep disturbances, which represents a departure from the existing knowledge base of sleep and pain in adolescents that draws heavily on pain populations. However, results should be interpreted with caution in light of the study’s limitations. First, the wide variety of sleep concerns makes it challenging to characterize the study’s population in terms of primary presenting sleep problems and which of those sleep concerns primarily drive relationships with pain. Future research should attempt to disentangle the relative contributions of behavioral versus organic sleep disorders. An additional limitation is our relatively small sample size which resulted in an inability to control for other important covariates. Furthermore, considering that the sample was based on a patient population of adolescents with sleep disturbances, some of which, but not all, endorsed experiencing chronic pain, the results may not be generalizable to adolescents with pre-existing chronic pain. Further, we do not have full information on the physical and mental health comorbidities (e.g., diabetes, ADHD) that characterize the sample and may influence results. Next, the measures used were brief, particularly for health behaviors and pain, as these were measured as secondary outcomes of the larger study. While a validated measure was used to measure pain, future research should consider more comprehensive measures that assess for the presence and severity of various types of pain (e.g., headache, joint pain). In addition, the use of objective measurement tools such as actigraphy or accelerometry is recommended for sleep, physical activity, and sedentary behavior. Furthermore, given the nature of the measures used, the present study does not explore whether or not meeting the recommended guidelines for movement behaviors is consequential for youth presenting with sleep disturbances who may be experiencing co-morbid pain. Future research should examine whether meeting the recommended guidelines for movement behaviors has a significant impact on sleep and/or pain concerns among youth. Lastly, the cross-sectional nature of the present study makes it impossible to discern causality among the variables of interest. Longitudinal studies are needed to elucidate the impact of improved sleep on pain, and whether these effects remain stable over time.

## Conclusion

The present study examined the relationship between insomnia and pain in a sample of adolescents presenting with sleep concerns. Findings replicated prior research suggesting high rates of pain in adolescents with a sleep disturbance. While poor sleep was found to be associated with worse pain, physical activity and screen time were not related to pain nor were they found to influence the sleep-pain relationship. Clinical awareness of the high comorbidity of insomnia and pain complaints should be used to guide the delivery of insomnia treatment in order to prevent the development or exacerbation of pain symptomatology. Finally, future research is needed to replicate these findings using more methodologically rigorous designs with pain as the primary outcome.

## Data Availability

Available upon request.

## Code Availability

Available upon request.
